# Influence of Orthotropy on Biomechanics of Peri-Implant Bone in Complete Mandible Model with Full Dentition

**DOI:** 10.1155/2014/709398

**Published:** 2014-11-03

**Authors:** Xi Ding, Sheng-Hui Liao, Xing-Hao Zhu, Hui-Ming Wang

**Affiliations:** ^1^Department of Stomatology, First Affiliated Hospital of Wenzhou Medical University, Wenzhou 325000, China; ^2^School of Information Science and Engineering, Central South University, Changsha 410083, China; ^3^Department of Oral and Maxillofacial Surgery, First Affiliated Hospital of Zhejiang University, Hangzhou 310006, China

## Abstract

*Objective.* The study was to investigate the impact of orthotropic material on the biomechanics of dental implant, based on a detailed mandible with high geometric and mechanical similarity. *Materials and Methods.* Multiple data sources were used to elaborate detailed biological structures and implant CAD models. In addition, an extended orthotropic material assignment methodology based on harmonic fields was used to handle the alveolar ridge region to generate compatible orthotropic fields. The influence of orthotropic material was compared with the commonly used isotropic model and simplified orthotropic model. *Results.* The simulation results showed that the values of stress and strain on the implant-bone interface almost increased in the orthotropic model compared to the isotropic case, especially for the cancellous bone. However, the local stress concentration was more obvious in the isotropic case compared to that in orthotropic case. The simple orthotropic model revealed irregular stress and strain distribution, compared to the isotropic model and the real orthotropic model. The influence of orthotropy was little on the implant, periodontal ligament, tooth enamel, and dentin. *Conclusion.* The orthotropic material has significant effect on stress and strain of implant-bone interface in the mandible, compared with the isotropic simulation. Real orthotropic mechanical properties of mandible should be emphasized in biomechanical studies of dental implants.

## 1. Introduction

Osseointegrated dental implants have been increasingly used to restore the masticatory function for edentulous and partially edentulous situations, including the case when only a single tooth is missing. Due to lack of the periodontal ligament, osseointegrated implants, unlike natural teeth, react biomechanically in a different fashion to occlusal force. It is, therefore, believed that dental implants may be more prone to occlusal overloading, which is often regarded as one of the potential causes for peri-implant bone loss and failure of the implant/prosthesis. As a numerical method for structure analysis that is suitable for complex biological structures, the finite element (FE) analysis has been widely used in dental biomechanics to evaluate the effect of various parameters, for example, implant geometry, prosthesis design, and stress and strain distribution in the peri-implant region.

Conversely, the validity of the FEM results is primarily dependent upon three types of modeling factors: the geometric similarity to the real structure, the material similarity, and the effectiveness of boundary conditions [1]. Among these factors, the boundary conditions can be modified interactively. By applying a new set of boundary conditions and reexecuting the FEM software, one can readily obtain a new set of simulation results.

Geometric modeling, however, is not a trivial task when one requires biological structures of high geometrical similarity, as well as detailed CAD implant models. In recent years, most dental studies constructed 3D models based on CT scan. While the tooth root has similar bone density with the mandible in which it is embedded, the entire single tooth model is difficult to extract [2], and the tooth crown usually lacks local feature details. Moreover, the biological soft tissues, such as the temporomandibular joint disc, articular cartilage, and periodontal ligament, are difficult to distinguish in CT scan. As a result, most previous studies utilized simplified mandibular models without refined biological structures.

The assignment of proper material properties is also a fundamental step to ensure predictive accuracy. The accurate modeling of biological tissues, such as bone related organs, is a difficult task because of their inherent nonhomogeneous and anisotropic characteristics. The heterogeneity is, in a certain sense, “directly” accessible based on CT Hounsfield Units [3]. Unfortunately, this is not the case for the anisotropy. An isotropic material has the same properties in every direction; in contrast, anisotropic material has different physical properties depending on spatial orientation. Among the variety of anisotropic materials, of the greatest practical value, are the so-called orthotropic and transversally isotropic materials. An orthotropic material has three mutually orthogonal axes of rotational symmetry so that its mechanical properties are different along each axis. A transversely isotropic material has one axis of symmetry that is normal to a plane of isotropy. Most previous studies adopted oversimplified isotropic material. Several authors incorporated transversely isotropic or simplified orthotropy into their models, but built only a limited segment of bone diaphysis, whose longitudinal axis is almost on the same straight line [4, 5], which is convenient to define one global orientation system. Bonnet et al. divided the entire mandible into four zones, with a specific local coordinate system for each one, to build their simplified orthotropic model [6]. However, with the curved longitudinal axis and irregular cross-sectional shape of the mandible, the orthotropic principal axes of longitudinal, radial, and tangential (circumferential) directions change from point to point inside the bone tissues, which is obviously not enough represented by only one or several local coordinate systems.

Recently, Liao et al. investigated a convenient methodology for the physical modeling of bone tissues [7], which creates longitudinal and radial volumetric harmonic fields to generate the orthotropic principal axes fields. The generation of harmonic fields needs only interaction of selecting point on the tips of condyle process and coronoid process, and the computation of principal axes from harmonic fields is stable; however, the method is only suitable for the edentulous mandible and not the dentate mandible. As the scalar distribution pattern of the harmonic field tends to conform well to the shape of object, it cannot generate a compatible longitudinal direction field in the alveolar ridge region of the mandible.

The objective of this study was to construct a high-quality dentate mandible model with detailed biological structures and integrate an accurate CAD model of ITI endosseous implant in the first molar region. Furthermore, the orthotropic methodology using harmonic fields was extended to handle the alveolar ridge region of the dentate mandible to generate compatible orthotropic fields. The influence of orthotropic material on the biomechanical behavior of mandible under masticatory loading was compared with the commonly used isotropic model and the simplified orthotropic model [6], especially in areas of bone surrounding the implant and natural teeth.

## 2. Materials and Methods

### 2.1. Model Designs

In order to obtain reliable numerical simulations, the morphometric characteristics of the mandible and the implant system must be precisely reconstructed.

In this study, three different types of data were collected to elaborate detailed mandible structures. First, both CT scan (SOMATOM SENSATION 64 Multi-Slice CT scanner) and MRI scan (GE 1.5T MRI scanner) of a dentate mandible and temporomandibular joint (TMJ) of a 28-year-old man were used to produce 410 CT image slices and 65 MRI image slices in DICOM format. Scanned images were imported into the ad hoc medical image processing and simulation software USIS (Universal Surgical Integration System). The initial 3D solid mandible (including the separation between cortical and cancellous bone) and parts of the temporal bone models were generated from CT scan data, and the solid models of the glenoid fossa, condyle, and articular disk were built from the MRI scan data. Then, a 3D registration procedure based on the fossa and condyle regions was performed to transform the articular disk model of MRI data into the coordinate system of CT data.

Following, a laser scan digitizer was employed to collect geometric models of a group of standard plaster cast teeth. Based on a hybrid method using shape prior to level-set and TPS transformation [2], each standard tooth was used to create a best-fit geometric model of the patient-specific tooth on CT, capturing the smooth tooth root as well as local details of tooth crown. These individual models were assembled and a Boolean operation was performed to generate the alveolar ridge in mandible bone. According to anatomical data from the literature [8], the separation surface between tooth enamel and dentin was built. The same procedure was applied to generate a 2 mm thick layer of TMJ articular cartilage on the fossa and condyle regions and an average thickness of 0.25 mm layer of periodontal ligament around each tooth root.

**Figure 1:**
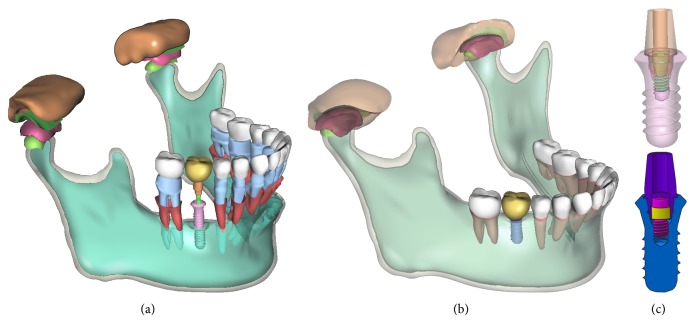
The 3D assembled solid model of complete dentate mandible.

Afterward, a real ITI endosseous implant system (Institut Straumann A.G., Switzerland) was split into components of abutment, implant fixture, internal connecting screw, and internal support ring, whose geometric models were obtained by laser scan. The implant system consists of implant fixture (10.0 mm in length and 4.8 mm in diameter, wide neck design), internal connecting screw, internal support ring, and abutment. These models were postprocessed and recovered with sharp edge features [9] and assembled to form an accurate CAD model of implant system, as shown in Figure 1(c). The implant system was placed in the first molar region of the mandible on right site, with a zirconia ceramics crown prosthesis model assembled on the implant abutment.

The final assembled solid model is presented in Figure 1, including the cortical and cancellous bone of the mandible, tooth enamel, dentin, periodontal ligament, temporal fossa, TMJ articular disc, temporal cartilage and condylar cartilage, endosseous implant fixture, internal connecting screw, internal support ring, and abutment and tooth crown prosthesis.

### 2.2. Material Properties

These solid models were imported into a self-developed biomedical modeling program to generate the FE volumetric mesh model, with adaptive mesh size that is optimal in the regions of biomechanical significance. Additionally, to model the TMJ joint, two interface contact pairs were generated in the disc-fossa cartilage interface and disc-condylar cartilage interface, as well as a group of capsular ligaments. A friction coefficient of 0.0001 was considered for these contact surfaces because of the existence of synovial fluid [10].

Then, three comparing models with different material properties were built to study the influence of elastic orthotropy on the dentate mandible. In all these models, tooth enamel, dentin, articular cartilage, implant, and crown prosthesis were considered to be isotropic, homogenous, and linearly elastic [11–13]: *E* = 84.1 GPa and *ν* = 0.33 for tooth enamel, *E* = 18.6 GPa and *ν* = 0.31 for dentin, *E* = 0.8 GPa and *ν* = 0.35 for articular cartilage, *E* = 103.4 GPa and *ν* = 0.35 for titanium implant, and *E* = 200 GPa and *ν* = 0.31 for tooth crown prosthesis of zirconia ceramics material. The TMJ articular disc was coupled with the mechanical properties of a silicone rubber using a multilinear model with a Poisson's ratio of 0.45 [12], and the periodontal ligament was coupled with a nonlinear viscoelastic model [13].

With regard to mandibular cortical and cancellous bone properties, the first FE model corresponded to a rough approximation commonly employed in the literature, regarding bone as an elastic, isotropic medium. The second model considered bone tissues as elastic and orthotropic continua. Nine independent constants must be used in the orthotropic case to reproduce the material symmetry with respect to two perpendicular planes [14], as shown in Table 1. To compare the results, their effective isotropic elastic modulus, *E* = 16.42 GPa and *ν* = 0.32 for cortical bone and *E* = 0.482 GPa and *ν* = 0.26 for cancellous bone, were deduced using the uniform strain and uniform stress isotropic bounds of Voigt and Reuss to describe the behavior of the first isotropic model.

**Figure 2:**
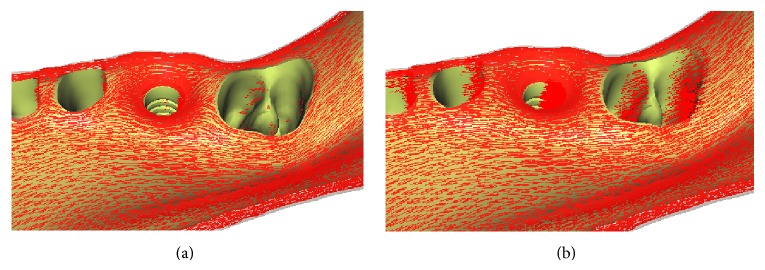
(a) Longitudinal axis vector field generated without virtual finite element. (b) Longitudinal axis vector field of alveolar ridge region generated using virtual finite elements.

As described by O'Mahony et al. [15], orthotropy is not in itself a problem for FEM. However, the curved longitudinal axis and irregular cross-sectional shape of the mandible do not easily lend themselves to the use of orthotropic material, for which the principal symmetry axes change from point to point inside the bone tissues. Although the orthotropic methodology using harmonic fields works well for the edentulous mandible [7], when there are teeth on the mandible, the direction of the longitudinal field of mandibular bone near the alveolar ridge region is not compatible with the real trajectory of maximum material stiffness, as shown in Figure 2(a).

To address this problem, we filled the alveolar ridge region of the mandible with virtual finite elements and then employed the harmonic methodology to generate the orthotropic principal axes fields [7]. Then, the local orthotropic material coordinate system of each element in the cortical and cancellous bone of mandible was generated, while ignoring these virtual finite elements, as shown in Figure 2(b).

To provide additional comparison, the third FE model used the simplified orthotropic modeling scheme of Bonnet et al. [6] and divided the mandible into four zones, with a specific local coordinate system for each one.

### 2.3. Boundary Conditions

**Figure 3:**
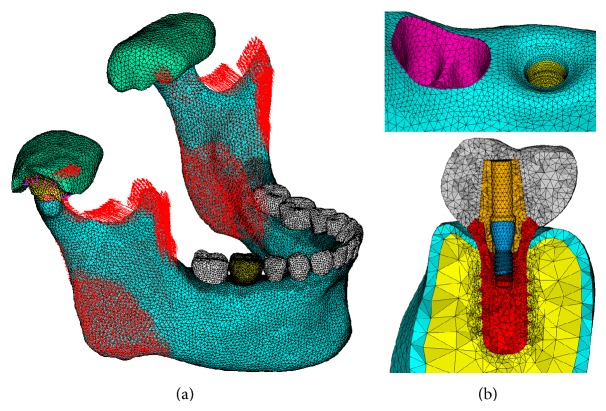
(a) FE model of dentate mandible with muscular force. (b) Local view of alveolar ridge region, and section view of implant/prosthesis system.

Sites for attachment of masticatory muscles were defined according to anatomical literature, on which the force from the following muscles was modeled with physiological muscular directions and load magnitudes: superficial and deep masseter; anterior, middle, and posterior fascicles of temporalis; and medial and lateral pterygoid [16], as shown in Figure 3(a).

These mandible models were then used to simulate the biting task in the intercuspal position. The occlusal surface was restrained from movement at occlusal direction. Restraints were also placed bilaterally at the endosteal surfaces of the temporal bones [16]. Both sides of the condyle's anterior incline were constraint to prevent rigid mandible displacement. Perfect bonding was assumed at the implant-bone interface and the implant-prosthesis interface.

## 3. Results

**Figure 4:**
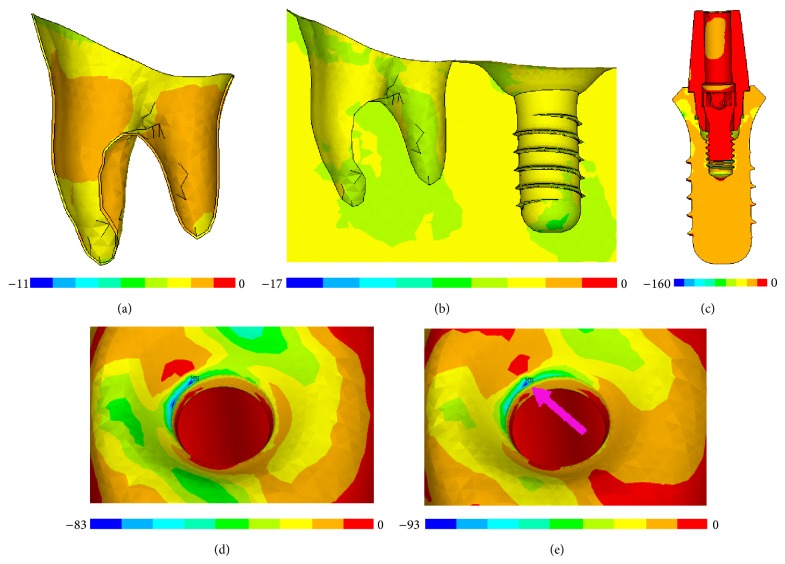
Contour plot of maximum compressive stresses. (a) Periodontal ligament of molar in orthotropic model; (b) cancellous bone in orthotropic model; (c) implant in orthotropic model; (d) cortical bone in orthotropic model; (e) cortical bone in isotropic model.

The simulation results demonstrated that the values of stress and strain almost increased in the orthotropic model compared to the isotropic case, both in the area of cortical bone and cancellous bone. But in the simple-orthotropic case, the values of stress and strain were irregular, compared to the realistic orthotropic case as well as isotropic case. There was little effect of orthotropy on the stress and strain values of implant, abutment, crown, periodontal ligament, tooth enamel, and dentin, compared with bone. For all comparing models, the values of equivalent, tensile, and compressive stress on the cortical bone surrounding implant were greater than those of adjacent molar and premolar. The stress mainly concentrated on the neck of cortical bone surrounding the implant. It should be noted that the local stress concentration phenomenon was more significant in the isotropic model compared to the orthotropic model, as shown in Figure 4.

In this study, we focused on the stress and strain of cortical bone and cancellous bone surrounding implant. Because the osseointegrated implant, unlike natural teeth, reacts biomechanically in a different fashion to occlusal force; this study focused on stress and strain analysis, including Von Mises stress and strain (equivalent stress and strain), principal tensile stress and strain, and principal compressive stress and strain in regions surrounding implant, as well as molar and premolar teeth.

### 3.1. Stress Variation on Cortical and Cancellous Bone Surrounding the Implant

**Figure 5:**
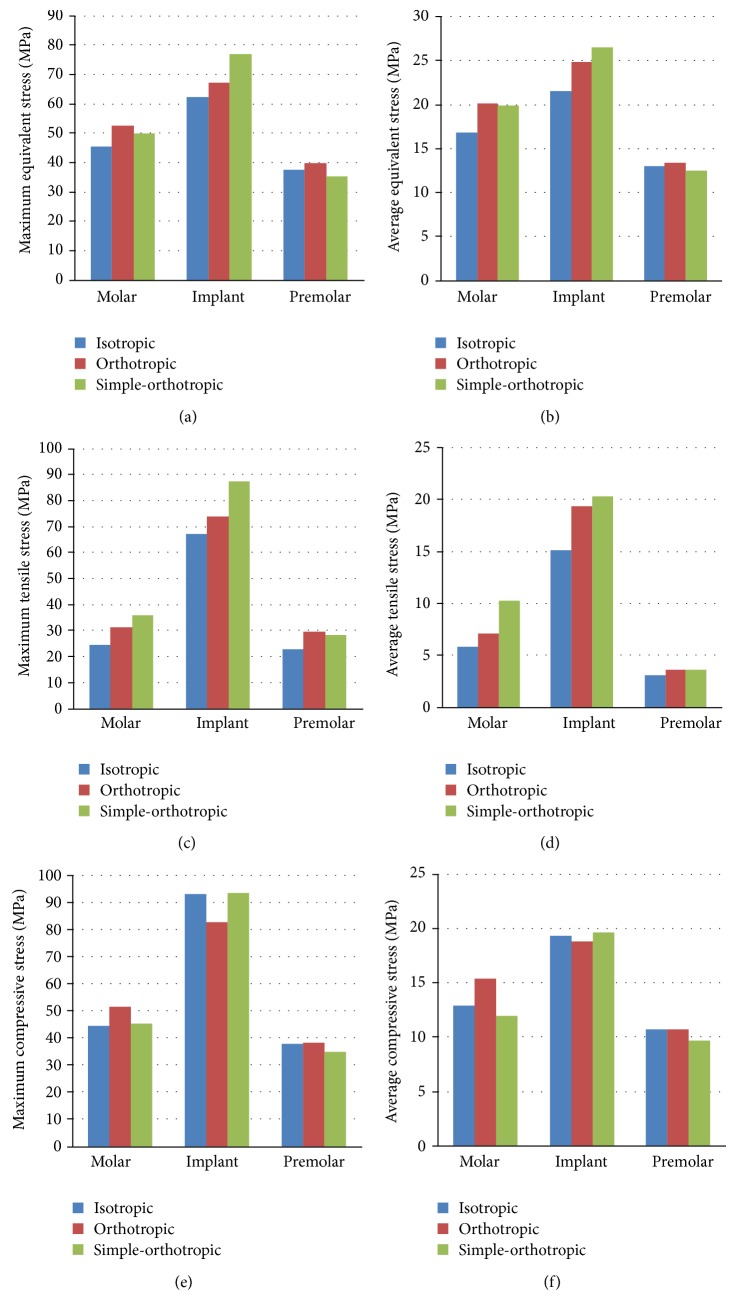
Stress distribution on the cortical bone surrounding the implant in the isotropic, orthotropic, and simple-orthotropic model ((a) maximum values of equivalent stress, (b) average values of equivalent stress, (c) maximum values of tensile stress, (d) average values of tensile stress, (e) maximum values of compressive stress, and (f) average values of compressive stress).

**Figure 6:**
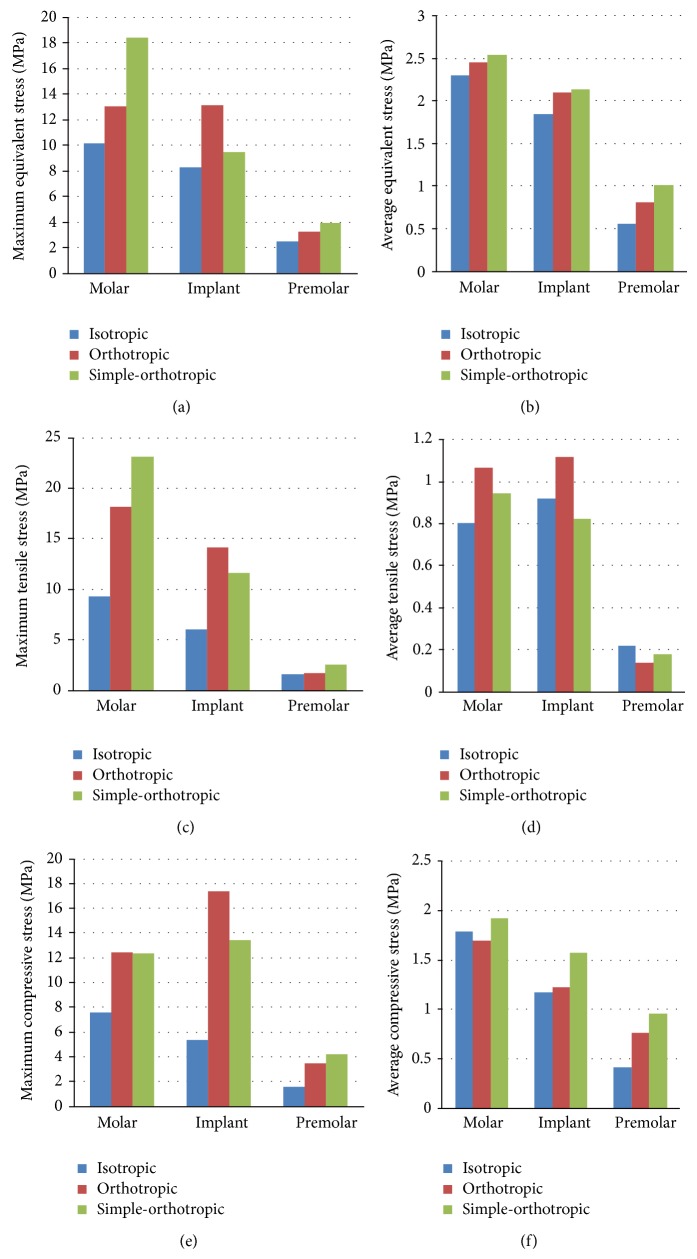
Stress distribution on the cancellous bone surrounding the implant in the isotropic, orthotropic, and simple-orthotropic model ((a) maximum values of equivalent stress, (b) average values of equivalent stress, (c) maximum values of tensile stress, (d) average values of tensile stress, (e) maximum values of compressive stress, and (f) average values of compressive stress).

The values of equivalent stress, tensile stress, and compressive stress on the cortical bone surrounding implant were greater than those of adjacent molar teeth and premolar teeth (Figure 5). The stress mainly concentrated on the neck of cortical bone surrounding the implant for all models. And the maximum and average values of equivalent stress, tensile stress, and compressive stress on the cortical bone were many times greater than those of cancellous bone. The orthotropic model would induce higher stress values than the isotropic model on the cortical and cancellous bone surrounding implant with few exceptions; but the simple-orthotropic model would induce higher values in some regions and get lower values in other regions than the real orthotropic model and the isotropic model (Figures 5 and 6).

The maximum values of equivalent stress, tensile stress, and compressive stress on the cortical bone surrounding implant increased 8.1%, 10%, and 13.2% in orthotropic model compared to isotropic model, while the average values of those increased 15.3%, 28.5%, and 8% in orthotropic model, respectively. The maximum values of equivalent stress, tensile stress, and compressive stress on the cortical bone surrounding implant increased 58%, 136.2%, and 226.7% in orthotropic model compared to isotropic model, while the average values of those increased 13.8%, 22%, and 4.5% in orthotropic model, respectively. Comparing orthotropic model with isotropic model, stress values on the cancellous bone around implant increased more obviously than those on the cortical bone, especially for maximum values.

### 3.2. Strain Variation on Cortical and Cancellous Bone Surrounding the Implant

**Figure 7:**
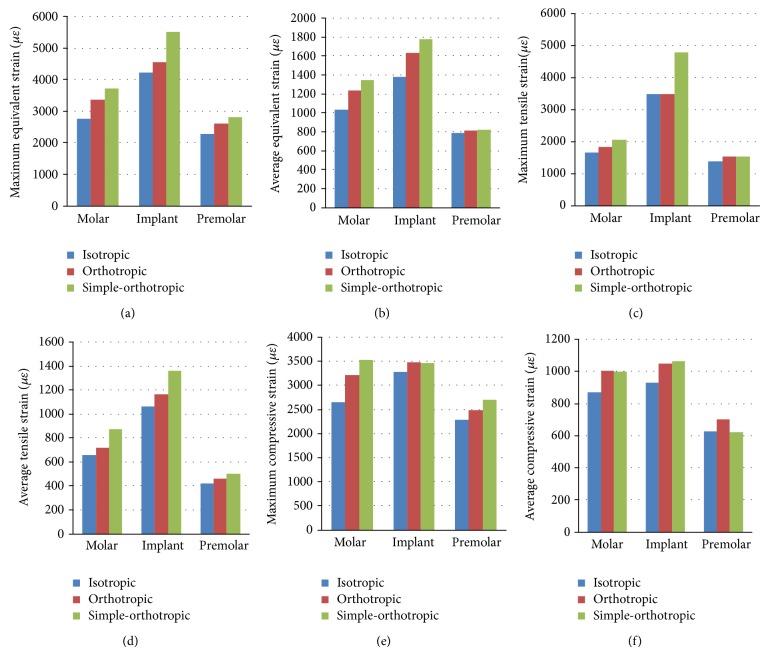
Strain distribution on the cortical bone surrounding the implant in the isotropic, orthotropic, and simple-orthotropic model ((a) maximum values of equivalent stress, (b) average values of equivalent stress, (c) maximum values of tensile stress, (d) average values of tensile stress, (e) maximum values of compressive stress, and (f) average values of compressive stress).

**Figure 8:**
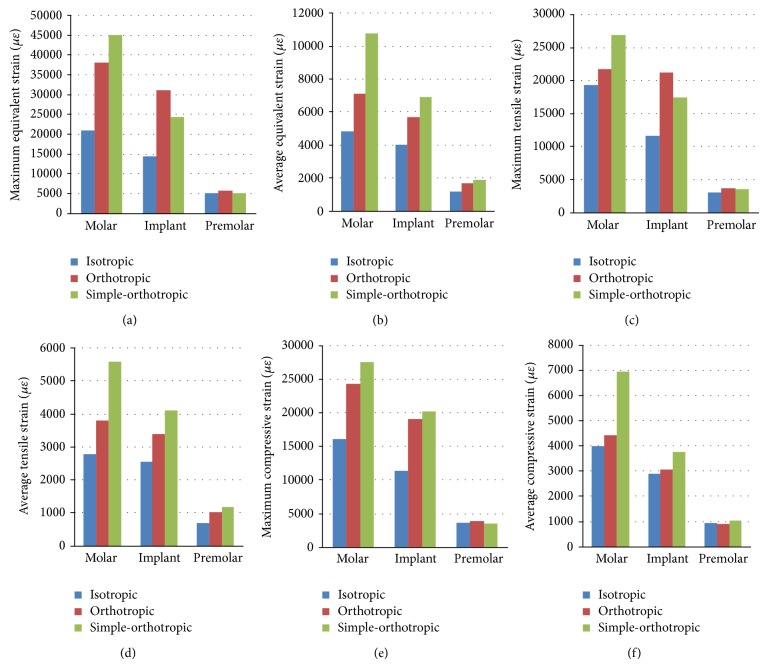
Strain distribution on the cancellous bone surrounding the implant in the isotropic, orthotropic, and simple-orthotropic model ((a) maximum values of equivalent stress, (b) average values of equivalent stress, (c) maximum values of tensile stress, (d) average values of tensile stress, (e) maximum values of compressive stress, and (f) average values of compressive stress).

The values of equivalent strain, tensile strain, and compressive strain on the cortical bone surrounding implant were greater than those of adjacent molar teeth and premolar teeth (Figure 7). The strain mainly concentrated on the bottom of cancellous bone surrounding the implant for all models. And the maximum and average values of equivalent strain, tensile strain, and compressive strain on the cancellous bone were many times greater than those of cortical bone. Orthotropic model would induce higher strain values compared to the isotropic model on the cortical and cancellous bone surrounding implant with few exceptions; but the simple-orthotropic model would induce higher values in some regions and get lower values in other regions compared to the real orthotropic model and the isotropic model (Figures 7 and 8).

The maximum values of equivalent strain, tensile strain, and compressive strain on the cortical bone surrounding implant increased 7.6%, 0.4%, and 5.9% in orthotropic model compared to isotropic model, while the average values of those increased 18.2%, 9.7%, and 13.3% in orthotropic model, respectively. The maximum values of equivalent strain, tensile strain, and compressive strain on the cortical bone surrounding implant increased 115.4%, 82.8%, and 67.4% in orthotropic model compared to isotropic model, while the average values of those increased 42.1%, 31.9%, and 5.8% in orthotropic model, respectively. Comparing orthotropic model with isotropic model, strain values on the cancellous bone around implant increased even more significantly than those on the cortical bone, especially for maximum values.

## 4. Discussion

The osseointegrated dental implant plays a role similar to that of natural teeth as it is exposed to static and dynamic loadings continuously. However, the transmission of functional forces to jaw bone via implant supported prosthesis is probably quite different from that via natural teeth with a healthy periodontium. The use of finite element physical modeling in dental biomechanics has significantly increased during the last decades, while many previous studies were compromised by oversimplifications of mandibular/implant geometry and material properties. It should be noted that the validity of simulation depends on assumptions made in modeling geometry, material properties, boundary conditions, and the bone-implant interface [17].

This paper introduced a high-quality dentate mandible model with detailed biological structures, taking advantage of multiple data sources. This mandible model consists of tooth enamel and dentin, periodontal ligament, complete temporomandibular joints, cortical bone, and cancellous bone and further integrates an accurate CAD model of ITI implant/prosthesis system in the first molar region. Meanwhile, the models were constrained from the masticatory muscles to simulate the actual situation of human biting and chewing. Some previous dentate mandible studies did not distinguish the tooth enamel and dentin or lacked periodontal ligament, which leads to the transmission of functional forces to mandible bone in a different way and influences the stress and strain distribution pattern. In contrast, this physical model more closely resembles the normal physiological structure of the natural teeth, as well as integrating an accurate implant model, which results in more reliable comparison between the implant system and natural teeth biomechanics. Some mandible studies did not build the temporomandibular joints, fixed the condyle process and coronoid process, and directly applied loading on the teeth, which could not simulate the real physiological biting and chewing action. In contrast, the physical model in this paper built TMJ articular disc, temporal cartilage and condylar cartilage, and contact interface surface, fixed the temporal fossa, and simulated chewing muscle attachments. All these settings increase the mandible mechanical similarity and should improve the accuracy of the finite element calculation.

In addition to the geometric similarity, information on the orientation of material axes is especially important, as local anisotropic behavior and regional variations have pronounced effects on the relationship between stress and strain patterns [18]. Some authors tried to simulate orthotropy by extensive manual work. Apicella et al. divided entire mandibular cortical bone into 62 sites [12], based on the orthotropic measurements [14]. Wirtz et al. cut cadaveric femurs into 2 mm slices, determined the principal directions using the orientation of the trabecular structures and the Haversian system [19]. Baca et al. stained the femurs to depict the direction of Haversian systems; repeated grinding exposed deeper layers and canal networks [20]. These methods are obviously not suitable for clinical analysis for patient-specific models.

Several authors incorporated simplified orthotropy into their models, using only one or several local coordinate systems [4–6]. However, with the curved longitudinal axis and irregular cross-sectional shape of the mandible, the orthotropic principal axes change from point to point inside the bone tissues, which is not fully represented by only a few local systems. The third comparing model of simple orthotropy in this study revealed irregular stress and strain distribution and verified this problem.

In this study, the simulation results of the isotropic and orthotropic model both demonstrated that the stress mainly concentrated on the neck of cortical bone surrounding the implant, and the strain mainly concentrated on the bottom of cancellous bone surrounding the implant. It agreed with other FE investigations that the occlusal forces are distributed primarily to the crestal bone, rather than evenly throughout the entire surface area of the implant interface [17, 21]. However, significant discrepancies of values are obtained between the results of isotropic and orthotropic simulations. Changes in geometry and orthotropic material properties could induce significant changes in strain patterns [22]. Bonnet et al. reported that significant differences in stress, strain, and strain energy densities were found in the comparison of isotropic and orthotropic models. Molar position was revealed to be the most critical one, from a stress and strain level point of view, for peri-implant bone [6].

In our study, it demonstrated that the values of stress and strain on the interface of implant bone almost increased in orthotropic case compared to isotropic case, both in the area of cortical and cancellous bone. In addition, the percentage increases in maximum stresses of cortical bone surrounding implant in the orthotropic case were up to 13.2% compared to those in the effectively isotropic model, but those were up to 226.7% on the cancellous bone surrounding implant. They were compressive stresses (the third principal stresses). The average stresses of cortical bone increase 28.5% at most and those of cancellous bone increase 22% at most. They were tensile stresses (the first principal stresses). The percentage increases in maximum strains of cortical bone surrounding implant in the orthotropic case were up to 7.6% compared to those in the effectively isotropic model but those were up to 115.4% on the cancellous bone surrounding implant. The average strains of cortical bone increase 18.2% at most, and those of cancellous bone increase 42.1% at most. They were both equivalent strains (Von Mises strains). It seemed that cancellous bone is more sensible to orthotropic properties than cortical bone, especially for maximum values of stress and strain. In another study on anisotropy of peri-implant stresses, the analytic results demonstrated that significant differences in stress and strain were found in the comparison of isotropic and orthotropic models and reported that the percentage increases in stresses in the anisotropic case were up to 70% compared to those in the effectively isotropic model [23]. In the study of Shen et al., the orthotropic model compared to the effectively isotropic model showed increased maximum principal stresses of 75% to 114% under axial loading [24]. O'Mahony et al. claimed that anisotropy would further increase what were already high levels of stress and strain in the isotropic case by 20 to 30% in the cortical crest. In cancellous bone, anisotropy increased what were relatively low levels of interface stress in the isotropic case by three- to fourfold to exceed bond strength levels [15]. They were almost consistent with our results, but their results' increase was smaller than ours in orthotropic model. These differences may be caused by our complete dentate mandible model with full orthotropy for cortical and cancellous bone and higher biofidelity implant.

It should be noted that, the local stress and strain concentration was more significant in isotropic case than that in orthotropic case, and the orthotropic model demonstrated some kind of mechanical optimality of the mandible. It may be because the orthotropy is more close to human physiological complexity and system adaptability, and the biomechanics distribution is more advantageous to chew activities and a self-defense mechanism. In the present study, the stresses and strains calculated by the model are properly interpreted only at a macroscopic level. To determine stresses and strains within microscopic structures, such as individual trabeculae, Haversian systems, cement lines or osteonal reversal lines, the lamina dura, or the tissue formed within several mm of the implant surface, would require refinement of the model to include such microscopic structural details and is beyond the scope of the current study. Such details can be treated by the finite element method but then require material property and structural information of tissues at a microscopic scale. At the microscopic level, the precise constitutive relations of the tissue formed at the implant-bone interface are uncertain [25], as are the actual failure criteria of bone and of the interface. It is of interest to note that, once debonding has taken place, it is the interface geometry which needs to be realistically accounted for, whereas the realistic material properties of the interface material are of minor importance [26].

This study extended the orthotropic material methodology based on harmonic fields to generate compatible orthotropic fields for the dentate mandible, which is reliable and almost automatic [7]. With regard to the influence of orthotropic material on the implant-mandible complex, according to the results of the present study,

1. orthotropic model further increased what were already high levels of most of stresses in isotropic case, and the cancellous bone was more sensitive to orthotropic properties than cortical bone; it seems that many previous studies in dental biomechanics underestimated the predicted stress levels;
2. for all comparing models, the stresses of cortical bone surrounding implant were greater than adjacent teeth; in natural teeth, the periodontal ligament acts as an intermediate cushion element; in contrast, occlusal loads are transmitted directly to surrounding bone from the dental implant, which could lead to stress concentration and cause microfracture at the bone interface, loosening of components of implant system, and unwanted bone resorption;
3. it should be noted that the stress concentration was more obvious in the isotropic model than that the orthotropic model, and the orthotropic model demonstrated some sort of mechanical optimality of the mandible; perhaps it is because the orthotropy is more close to human physiological complexity and system adaptability, and the stress distribution is more advantageous to chew activities and a self-defense mechanism; it is also the result of the long-term evolution of biological structures.

## 5. Conclusion

Within the limitations of this study, we conclude that the true orthotropy has significant effects on stress and strain distribution and could improve the realism of simulations. It was determined that bone orthotropy cannot be neglected in numerical simulations for finite element analysis of dental implants.

## Figures and Tables

**Table 1:** Orthotropic elastic coefficients for compact (Comp.) and cancellous (Canc.) bone of the dentate mandible^ (1)^.

	*E*_1_	*E*_2_	*E*_3_	*G*_12_	*G*_13_	*G*_23_	*ν*_12_	*ν*_13_	*ν*_23_
Comp.	**12.7**	**17.9**	**22.8**	**5.0**	**5.5**	**7.4**	**0.18**	**0.31**	**0.28**
Canc.	0.511	0.114	0.907	0.078	0.434	0.081	0.22	0.31	0.30

^(1)^ *E*_*i*_ represents Young's modulus (GPa); *G*_*ij*_ represents shear modulus (GPa); *ν*_*ij*_ represents Poisson's ratio. The 1st direction is radial, the 2nd direction is tangential (circumferential), and the 3rd direction is axial (longitudinal).
